# Influence of Multi-Modal Warning Interface on Takeover Efficiency of Autonomous High-Speed Train

**DOI:** 10.3390/ijerph20010322

**Published:** 2022-12-25

**Authors:** Chunhui Jing, Haohong Dai, Xing Yao, Dandan Du, Kaidi Yu, Dongyu Yu, Jinyi Zhi

**Affiliations:** 1 Department of Industrial Design, School of Design, Southwest Jiaotong University, Chengdu 610031, China; 2 School of Mechanical and Aerospace Engineering, Nanyang Technological University, Singapore 639798, Singapore

**Keywords:** multi-modal interface, autonomous driving, high-speed train, takeover efficiency

## Abstract

As a large-scale public transport mode, the driving safety of high-speed rail has a profound impact on public health. In this study, we determined the most efficient multi-modal warning interface for automatic driving of a high-speed train and put forward suggestions for optimization and improvement. Forty-eight participants were selected, and a simulated 350 km/h high-speed train driving experiment equipped with a multi-modal warning interface was carried out. Then, the parameters of eye movement and behavior were analyzed by independent sample Kruskal–Wallis test and one-way analysis of variance. The results showed that the current level 3 warning visual interface of a high-speed train had the most abundant warning graphic information, but it failed to increase the takeover efficiency of the driver. The visual interface of the level 2 warning was more likely to attract the attention of drivers than the visual interface of the level 1 warning, but it still needs to be optimized in terms of the relevance of and guidance between graphic–text elements. The multi-modal warning interface had a faster response efficiency than the single-modal warning interface. The auditory–visual multi-modal interface had the highest takeover efficiency and was suitable for the most urgent (level 3) high-speed train warning. The introduction of an auditory interface could increase the efficiency of a purely visual interface, but the introduction of a tactile interface did not improve the efficiency. These findings can be used as a basis for the interface design of automatic driving high-speed trains and help improve the active safety of automatic driving high-speed trains, which is of great significance to protect the health and safety of the public.

## 1. Introduction

Urban public transportation is a mobile public construction facility in the city. In recent years, public transport automatic driving has received widespread attention from academia and industry, of which the safety of automatic driving is directly related to public health. At present, the research into public transport automatic driving mainly focuses on automotive applications, and few studies investigate train drivers [1]. However, high-speed trains are an important means of land transportation [2]. As a large-scale public transport mode, the passenger capacity and operating capacity of high-speed rail are huge, so the driving safety of high-speed rail has a profound impact on public health.

At high speed, the braking distance of a high-speed train is very long (a 270 km/h train needs 3.7 km to stop) (Heping et al. 2003) [3]. However, humans’ driving reaction abilities are limited, and manual control of a high-speed train can lead to accidents caused by human error. Therefore, automatic driving technology has good application prospects in the field of high-speed trains [2]. In 2019, China built a 350 km/h Beijing–Zhangjiakou automatic high-speed train, thus realizing the engineering verification of automatic high-speed train technology.

Automatic driving technology also brings new train safety risks [4]. The current automatic high-speed train mainly adopts the automatic driving mode with a driver on duty. Similar to automatically driven cars, in special situations such as sudden danger or automatic driving failure, the automatic driving system will issue a takeover request (TOR) through the warning interface, requiring the driver to withdraw the control right of the vehicle as soon as possible and carry out the emergency disposal operation [4]. However, at present, the method of a manual takeover of the high-speed train autopilot is relatively simple (only a visual interface), so whether the driver can take over in the fastest possible time has become an exigent research question. In addition, train driving has its unique characteristics, cannot overtake another train, cannot leave the track, and travels faster than cars. Therefore, the driving information that the train driver has to deal with is different from that of the car driver [5], so it is difficult to apply the research conclusions on car interface performance directly to high-speed trains. As a result, it is necessary to carry out research on the warning interface performance of the high-speed train.

Due to the high-speed train involving more personnel, the consequences of a high-speed train accident are extremely grievous. For example, the derailment incident in Ashdod Town, Germany, and the particularly serious railway traffic accident on China’s Yongwen line both caused hundreds of casualties [4], highlighting the necessity of improving the performance of the warning interface of a high-speed railway. A multi-modal interface can make full use of the complementary attributes of drivers in multiple sensory modalities, and it has been proven to have better early warning efficiency than the visual interface in a car [6]. However, at present, the high-speed train warning interface has not integrated the application of a multi-modal interface, and there is a lack of related research. Based on this, the following research will be carried out:

(1) Compare and determine the modalities of the automatic driving interface for high-speed trains with the highest takeover performance;
(2) Provide optimization suggestions for the current high-speed train interface to improve the safety of automatic driving of high-speed trains.

## 2. Related Work

The current automatic high-speed train warning interface is a simple improvement based on the traditional train interface, which generally lacks research and critical inspection [5]. The research on multi-modal warning interfaces in automatic driving has mainly concentrated on automotive applications. The types of interface modalities mainly include visual, auditory, and tactile interfaces.

### 2.1. Application Status of Automatic High-Speed Train Interface

According to the “Interim overall technical scheme for ATO system of high-speed train” issued by the Ministry of Science, Technology, and Information Technology of China Railway Corporation [7] and the grade of automation (GOA) of rail transit in international standards [8], the automatic driving system of high-speed trains can be divided into four grades according to the automation level (Table 1).

**Figure 1:**
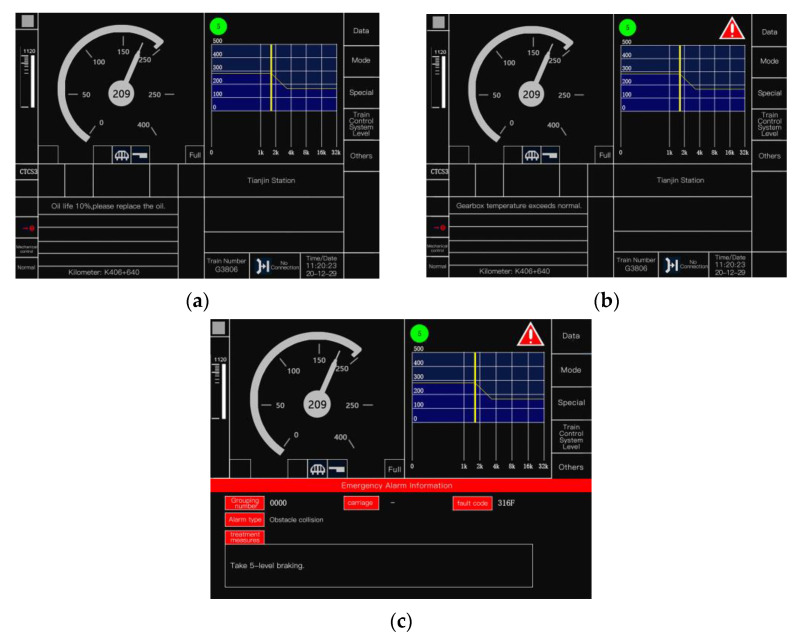
Warning interface of high-speed train ((**a**) level 1 warning interface; (**b**) level 2 warning interface; (**c**) level 3 warning interface).

At present, the automatic driving level of high-speed trains with application prospects is GOA2. According to the “Specification for the display ofman–machine interface (DMI) of CTCS-3 Train Control on-board equipment (v1.0)” [9], the current warning interface of GOA2 automatic driving high-speed train is mainly divided into three levels. The form of the warning interface is shown in Figure 1, and the interface warning method is shown in Table 2.

### 2.2. The Influence of Multi-Modal Interface on Takeover Efficiency

At present, the types of interface modalities used in the field of autonomous driving mainly include visual, auditory, and tactile interfaces, which are mainly used in the car. The following is a description of the current research status of the influence of each modal interface on the takeover performance.

#### 2.2.1. Research on the Impact of Visual Warning Interface on Takeover Performance

The visual interface is currently the most important form of the automatic driving takeover request interface. It usually sends out a warning by illuminating some visual elements (icons/area) on the interface [10]. Many studies have achieved improvements in takeover performance by optimizing the attributes of visual interface elements, for example, comparing the impact of different icon sizes and spacing on the performance of takeover [4,11,12]. In addition, since the driver cannot always focus their visual attention on the warning interface during driving, they may miss the warning information. Therefore, studies have tried to change the display position of the interface to increase the driver’s attention opportunities, such as using HUD technology to project the interface on the car window [13]. Another alternative is to change the form of the visual display, such as using LED strips or light strips to attract attention [14].

#### 2.2.2. Research on the Impact of Auditory Warning Interface on Takeover Performance

Compared with the visual interface, the auditory interface has the advantage of “no need to look at” [15] and does not occupy the driver’s visual attention resources. In addition, the content of the auditory signal is easily understood by people [16], which is helpful in improving processing efficiency. The audible warning interface has been used in some automatic driving systems, such as warning about obstacles when parking, or warning about surrounding vehicles with a risk of collision when driving [17]. The auditory takeover interface may take many forms, such as abstract (that is, nonverbal) warning sounds, such as beeps, or verbal warning sounds (divided, for example, into male and female voices). Some studies have compared the differences between a verbal auditory interface and an abstract auditory interface [18], and some other studies have also compared the differences in voices of different genders [19,20,21], as well as the differences between progressive and stable voice interfaces [22]. In summary, for the auditory alert interface, a variety of sound interface elements will have an impact on the takeover performance. Interface element characteristics, such as sound language abstraction, gender, gradualness–constancy, and other relevant characteristics, will have an impact on takeover variables such as takeover time and intelligibility. However, the research conclusions are not uniform, and there is a lack of relevant research on the auditory interface of high-speed trains.

#### 2.2.3. The Impact of Tactile Warning Interface on Takeover Performance

Compared with visual and auditory interfaces, the tactile interface is less used [23], but its application is growing rapidly [19,20]. At present, the tactile warning interface is mainly presented in the form of a vibration interface [24]. The advantage of the tactile interface is that it is not disturbed by the external environment (such as noise or visual occlusions). However, the disadvantage is that the amount of information transmitted is limited, which is not suitable for issuing complex alerts. At present, the main functionality of the vibration warning interface in the automatic driving system is supplied through a cushion, directional controller (such as steering wheel), seat back, or seat belt. The cushion is the most commonly used location [25,26].

#### 2.2.4. The Impact of the Multi-Modal Warning Interface on Takeover Performance

The integrated application of visual, auditory, and tactile interfaces can form a multi-modal interface. Some studies claim that, compared with single-modal interfaces, multi-modal interfaces can output more information from multiple modalities per unit of time, which can improve task efficiency, so they have greater research and application potential [27]. For example, De Groot et al. [28] found that an auditory–visual interface could provide left/right turn instructions more effectively than an auditory interface. Prewett et al. [29] found that the task completion efficiency (error rate, task completion time, and reaction time) of a visual–tactile interface was higher than that of a visual interface. Research has found that people are more likely to accept takeover requests from multimodal interfaces when it comes to sending reminders, which allows drivers to respond more quickly [30]. Bazilinsky et al. [31] found that drivers have the highest preference for warning interfaces that include all three modalities (auditory, tactile, and visual). However, other studies have found that multi-channel interface interaction is more urgent than single-channel interface interaction [32], which may lead to drivers’ nervousness and panic. In the field of aviation, Gauci J [33] found that it is more feasible for pilots to use a combination of touchscreen gestures and voice commands to control a variety of avionics systems. The current research conclusions on multi-modal takeover interface are not uniform, and there is a lack of research on automatic driving trains.

Based on the above research foundation, two specific research goals are proposed:

(1) Compare warning interface forms and determine the best multi-modal warning interface form in the situation of the emergency takeover of automatic driving high-speed trains (level 3 warning).
(2) Determine the interface elements and improvement directions that need to be optimized in the existing automatic driving high-speed train visual warning interface (level 1/level 2/level 3), and provide a reference for the optimization design of the high-speed train warning interface.

## 3. Research Method

### 3.1. Participants

A total of 48 subjects were selected for this experiment. The participants were not employed as high-speed train drivers, but they received a week’s training on simulated driving skills on the high-speed train. They had not received any training prior to this, and after the training, they all had a basic understanding of the rules of high-speed train driving, the meaning of railway signals and cab instrumentation, and how to operate the controls on the driving console. The criteria for verifying that the participants passed the driving simulation was that they drove the simulation three times for a minimum of one hour without any errors. This experimental approach to high-speed rail simulation was validated in a study by Guo et al. [2]. The age range of the participants was 20 to 32 years old, with a mean of 26.7 years old and SD = 5.5 years old, with the majority of them being students related to high-speed train modeling and design. The participants were divided into four groups of 12 participants each, corresponding to four types of multichannel interfaces. All participants had a normal or corrected vision and no color blindness.

### 3.2. Experimental Equipment

**Figure 2:**
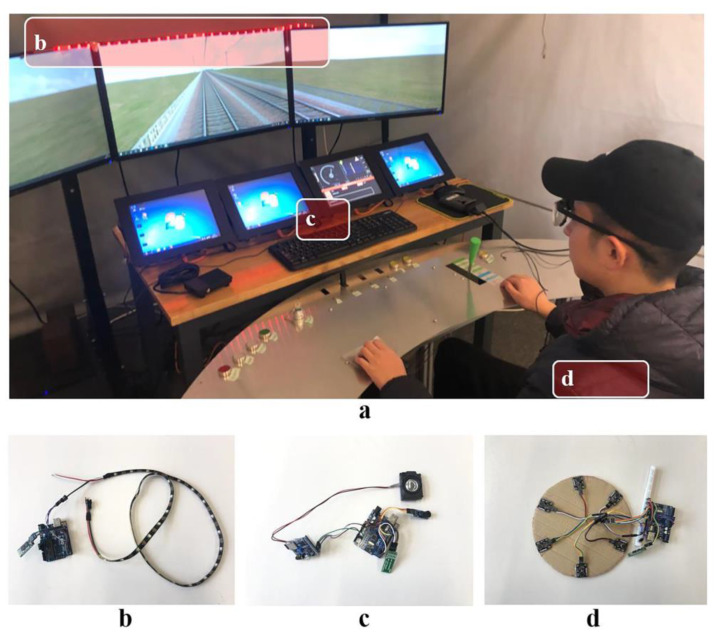
High-speed train simulation driving platform and multi-modal warning interface prototype ((**a**) high-speed train simulation driving platform; (**b**) visual interface prototype and action location; (**c**) visual interface prototype and action location; (**d**) tactile interface prototype and auction location).

A high-speed train simulation driving platform (Figure 2a) was used to simulate real high-speed rail driving at a ratio of 1:1, and three 32-inch LCD monitors were used to simulate driving vision. Equipped with OpenRail high-speed rail simulation driving software, it simulates the real conditions of the Beijing–Harbin Line (50 km long in the experimental section) at 350 km/h. Simulated weather conditions were good, the landscape and towns along the way were evenly distributed, and the traffic load was small. When the simulator was running, the frame rate of the dynamic image was locked to 60 Hz to ensure rapid changes in the image, and to present high-quality movies with a high resolution of 1920 pixels by 1080 pixels.

Tobii Glasses2 wearable eye tracker. Due to its lightweight, it can collect eye movement data such as gaze and saccades of the eyes in a completely natural state. The sampling frequency is 50 Hz, and it is driven by a desktop computer with the Windows 10 operating system installed.

Other equipment: a ThinkPad E14 notebook computer is used to run the Tobii eye tracker software; a desktop computer is used to control three different modalities of warning interactive prototypes; a digital video recorder to record the experiment process.

### 3.3. Prototype of Multi-Modal Warning Interface

In order to simulate a visual, auditory, and tactile warning interface, multi-modal warning interface prototypes were developed and implemented in the experiment together with the high-speed train simulation driving platform. Because the driver must use the visual interface during the driving of the train, four types of multi-modal warning interfaces that all include visual interfaces were developed in this research: (1) visual interface, (2) auditory–visual interface, (3) visual–tactile interface, and (4) auditory–visual–tactile interface. Correspondingly, the participants were also divided into four groups.

Since the level 3 warning is the most urgent in the experiment, the visual interface was adopted for the level 1 and level 2 warnings and the multi-modal interface was adopted when the participants face the level 3 warning.

#### 3.3.1. Visual Interface Prototype

The visual interface prototype was composed of two parts. The first part was the LED light strip, which is located in the cab window and flush with the driver’s head, and was mainly used to stimulate the driver’s visual sense immediately and strengthen the force of the remainder (Figure 2b). A 1 m long Ws28712b-5vled light belt was selected, with 30 LED beads built in. At present, the visual warning mode implemented in a typical high-speed train is a red indicator light. This is because, in human visual perception of light, red color has the longest wavelength and is most likely to attract people’s attention. In addition, studies have shown [34] that a strobe can improve the efficiency of information transmission, and the flashing frequency should be controlled 3–10 times/second, so red LED light combined with a 4 Hz strobe was used to strengthen the reminder.

#### 3.3.2. Auditory Interface Prototype

The auditory interface prototype was only activated at warning level 3 (Figure 2c). A passive buzzer was used to simulate a “beep” sound with a tone of 300 Hz and a frequency of 4 Hz. The passive buzzer and voice player driven by the Arduino UNO-R3 microcontroller to form an auditory interface prototype, which was arranged on the console facing the driver.

#### 3.3.3. Tactile Interface Prototype

The tactile interface prototype was only activated at warning level 3 (Figure 2d). Six linear resonant motors were evenly distributed on a circle with a radius of 150 mm and driven by an Arduino UNO-R3 microcontroller to form a tactile interface prototype with vibration reminders. It was placed on the seat.

#### 3.3.4. Takeover Information Interface Prototype

Consistent with the real high-speed train bridge, the takeover information interface prototype carried the warning interface information through four 8-inch screens. The main function was to inform the driver of the warning level and warning content and to prompt the driver to take measures. The takeover information interface was displayed in all warning levels.

### 3.4. Variables Design

The independent variable was the type of multi-modal interface, including four levels: (1) visual interface, (2) auditory–visual interface, (3) visual–tactile interface, and (4) auditory–visual–tactile interface.

Dependent variables included behavioral indicators and eye movement indicators. Behavioral indicators mainly consisted of the takeover time, because takeover time was the most direct indicator of takeover efficiency [35]. Eye movement indicators included two aspects: fixation indicators and saccade indicators. This was because, based on the research of Henderson et al. [36], eye movement behavior during scene browsing could be divided into two stages: fixation and saccade. At the same time, it was difficult for a single eye movement index to fully explain the eye movement characteristics in driving behavior. It was necessary to comprehensively consider and study multiple eye movement indexes. Fixation indicators were used to compare the takeover performance of the warning interface of levels 1 and 2 because these interfaces need to obtain takeover information through the screen and need to examine the driver’s visual focus. Saccade indicators were used to compare the takeover performance of different types of multi-modal interfaces at level 3. This was because the focus of the multi-modal takeover interface is to make the driver react to the stress immediately, so the saccade indicator was used to measure the driver’s response speed and urgency. The specific indicators are as follows:

#### 3.4.1. Takeover Time

The takeover time was a direct manifestation of the driver’s takeover efficiency, and it is also one of the most important indicators to evaluate the driver’s takeover performance. Takeover time was defined as the time that elapsed between when the warning signal was sent out and when the participant made the takeover action.

#### 3.4.2. Fixations

(a) First fixation duration. The first fixation duration was defined as the time elapsed between the sending of a warning message and the time when the participant first gazes at the screen interface (the so-called area of interest, AOI). This information was used to indicate the initial recognition of the target stimulus [37]. The shorter the first fixation duration, the stronger the target’s ability to attract attention [38]. It also showed that the message can be delivered more effectively to the audience.
(b) Total fixation duration. This indicator corresponded to the total fixation duration of the participant in the on-screen interface (AOI) during the takeover process. The total fixation duration can reflect the degree of cognitive difficulty. The longer the total fixation time, the higher the participant’s attention to the area, the greater the difficulty of the corresponding information processing, and the lower the processing efficiency [39].
(c) Fixation count. Fixation count of the participant in the on-screen interface (AOI) during the takeover time. The more fixation points, the more difficult it is to determine the target and extract information [38].

#### 3.4.3. Saccades

(a) Saccade count. This indicator reflects the count of visual saccades made during the takeover when receiving the three-level warning. The greater the saccade count, the longer the search process and the inability to determine the target position in time, which affects the takeover efficiency to a certain extent.
(b) Average saccade velocity/maximum saccade velocity. These indicators correspond to the average saccade velocity and maximum saccade velocity during the takeover process when the participant receives the level 3 warning. The larger the velocity, the faster the saccade and the more alert the participants.
(c) Average saccade amplitude/maximum saccade amplitude. These indicators reflect the average saccade amplitude and maximum saccade amplitude during the takeover process when the participant receives the level 3 warning. The larger the saccade amplitude, the more meaningful exploration of new areas or locations. When these indicators are large, the subject will also be considered nervous, potentially corresponding to poor recognition of the rail environment [40].
(d) First saccade duration. The duration of the first saccade during the takeover process when the participant received the level 3 warning. The longer the duration of the first saccade, the more difficult it is to process the task and the slower the response speed, resulting in higher takeover time and lower takeover efficiency [2].

### 3.5. Experiment Design

The experimental steps were as follows:

(1) Demographic information. Before the start of the experiment, participants were asked to fill in basic demographic information, including age, height, and weight.

(2) Training. We conducted high-speed train simulation driving training for participants to ensure that they are familiar with the high-speed train simulation driving platform and the use of experimental tasks.

(3) Eye tracker calibration and simulated experiment. We informed the participants of the purpose and process of the experiment and helped the participants wear the eye tracker and complete the eye tracker calibration. We tested the multi-modal interactive prototype and allowed users to respond when receiving signals from the prototype (such as lights, sounds, vibrations, and other target stimuli). Based on their input, we checked the content of the takeover task displayed on the screen and then made adjustments to calibrate the instrumentation. The simulation experiment took five minutes, during which participants will be asked to complete level 1–3 warning takeover tasks in random order.

(4) Experimental task. The entire simulated driving process lasted for 20 min. Participants first drove normally for five minutes. Then, during the driving process, level 1–3 warnings would appear randomly. Each level of warning would appear three times, for a total of 9 warnings. The mean values of the three experimental results were used as the final data. Among them, the first and second warnings only displayed the screen interface warning information. The level 3 warning not only had the screen interface information but was also accompanied by the corresponding multi-modal interaction combinations. Each group of participants only encountered one type of multi-modal interaction combination. If participants received an alert, they were required to immediately check the cause of the accident, follow disposal recommendations, and initiate a takeover as suggested with the highest efficiency. If there was an error, it would be marked as a failed task, and the task would be repeated until it succeeded.

(5) At the end of the experiment, we recorded the data and followed up with the participants.

## 4. Results

In order to analyze the impact of different multi-modal warning interactive interfaces on the efficiency of takeover in different warning levels, we first performed the Shapiro–Wilk normal test on the data of each dependent variable. If *p* < 0.05, we selected the independent sample Kruskal–Wallis test method to analyze the indicators. If *p* > 0.05, we used the one-way analysis of variance method to analyze.

### 4.1. Takeover Time

#### 4.1.1. Comparison of the Takeover Time of the Visual Warning Interface in Different Levels of Warning

The independent sample Kruskal–Wallis test method was used to compare and analyze the difference in takeover time under different warning levels (level 1 warning/level 2 warning/level 3 warning). Our analysis revealed that there were significant differences in the average takeover time of different warning levels (*p* < 0.001). In the pairwise comparison, it was found that the takeover time of the participants when facing the level 1 warning [1.863 (1.585–2.223)] was significantly less than the takeover time when facing the level 3 warning [2.794 (2.295–3.11)]. The takeover time under the level 2 warning [1.649 (1.468–2.01)] was significantly less than the takeover time under the level 3 warning. There was no significant difference between the takeover times of the level 1 warning and the level 2 warning, as shown in Table 3.

#### 4.1.2. Comparison of the Takeover Time of Each Multi-Modal Warning Interface in the Level 3 Warning

**Figure 3:**
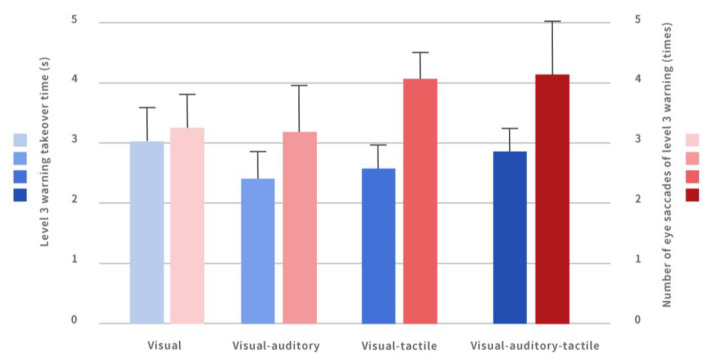
The takeover time and the number of eye saccades for level 3 warning interface under different warnings.

The one-way analysis of variance method was used to compare the impact of different multi-modal warning interfaces on participants’ takeover time under the level 3 warning, and significant differences were found (*p* = 0.047), as shown in Figure 3. Through multiple comparisons, it was found that the takeover time of participants under the visual–auditory warning interface (2.436 ± 0.425) was significantly less than that of participants under the purely visual interface (3.006 ± 0.647).

### 4.2. Fixations

#### 4.2.1. Comparison of the First Fixation Duration of the Visual Warning Interface in Different Levels of Warning

The independent sample Kruskal–Wallis test method was used to analyze the impact of different levels of warnings on the participants’ first fixation duration, and significant differences were found. Through pairwise comparison, it was found that the first fixation duration of the participant in the level 2 warning [267.5 (182.25–423.5)] was significantly less than that of the level 1 warning [586 (327.5–891.5)]; that the first fixation duration in the level 3 warning [368.5 (271–545.75)] was significantly less than that of the level 1 warning [586 (327.5–891.5)]; and that there was no significant difference in the first fixation duration of the participants between the level 2 and level 3 warnings, as shown in Table 3.

#### 4.2.2. Comparison of the Total Fixation Duration within the AOI of the Visual Warning Interface in Different Levels of Warnings

The independent sample Kruskal–Wallis test method was used to analyze the impact of different levels of warnings on the participants’ total fixation duration, and it was found that there were significant differences (*p* = 0.000). In the pairwise comparison, it was found that the total fixation duration of the participants in the level 1 warning [392 (322.75–527.5)] was significantly less than that of the level 2 warning [586 (327.5–891.5)]; that the total fixation duration of the participants under the level 1 warning [392 (322.75–527.5)] was significantly less than that of the level 3 warning [600.5 (426.75–854.25)]; and that the participants’ total fixation duration under the level 2 and 3 warnings suggested no significant difference in total fixation time within AOI, as shown in Table 3.

#### 4.2.3. Comparison of the Number of Fixations Count in the AOI of the Visual Warning Interface in Different Levels of Warning

The independent sample Kruskal–Wallis test method was used to analyze the influence of different levels of warnings on the number of fixations count of the participants. A significant difference was found (*p* = 0.001). Through pairwise comparison, it was found that the fixation count of the participants in the level 3 warning [1.67 (1.33–2)] was significantly less than that of the level 2 warning [2 (2–3)]; that the fixation count in the level 1 warning [2 (1–2.33)] was significantly less than that of the level 2 warning [2 (2–3)]; and that there was no significant difference in the fixation count between the participants in the level 1 warning and the level 3 warning, as shown in Table 3.

### 4.3. Saccades

#### 4.3.1. Comparison of Saccade Count under Different Warnings

A single-factor analysis of variance method was used to compare the effects of four multi-modal interactive combination warning methods on the saccade count during the takeover process in the level 3 warning, and it was found that there were significant differences (*p* = 0.000). Among them, the participants had the lowest saccade count under the auditory–visual warning interface (3.08 ± 0.85), and the highest saccade count under the auditory–visual–tactile interface (4.25 ± 0.90), as shown in Figure 3.

Through multiple comparisons, it was found that the saccade count involved in the purely visual interface (3.11 ± 0.63) was significantly lower than that in the visual–tactile interface (4.11 ± 0.72); that the saccade count involved in the purely visual interface (3.11 ± 0.63) was significantly less than that of the auditory–visual–tactile interface (4.25 ± 0.90); that the saccade count in the auditory–visual interface (3.08 ± 0.85) was significantly less than that in the auditory–visual–tactile interface (4.25 ± 0.90); and that the saccade count in the auditory–visual interface was significantly less than that in the visual–tactile interface.

#### 4.3.2. Comparison of Average Saccade Velocity/Maximum Saccade Velocity under Different Warnings

**Figure 4:**
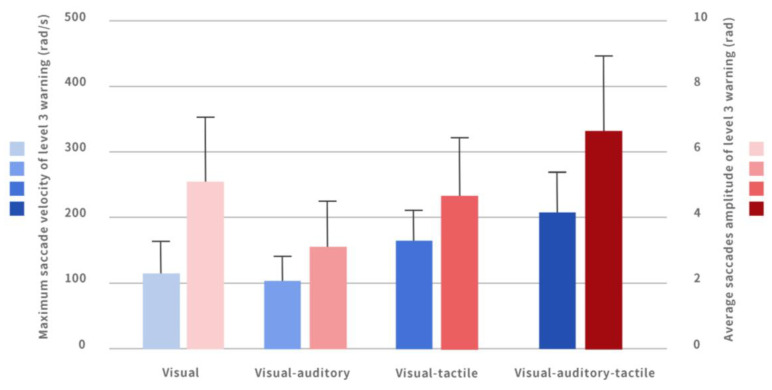
Maximum saccade velocity and average saccade amplitude of level 3 warning interface under different warnings.

A single-factor analysis of variance method was used to compare the effects of four multi-modal interactive combination warning methods on the average saccade velocity/maximum saccade velocity of participants during the takeover in the level 3 warning. The results showed that there was no significant difference in the average saccade velocity; there was a significant difference in the maximum saccade velocity (*p* = 0.000), as shown in Figure 4.

Through multiple comparisons after ANOVA, the participants’ maximum saccade velocity under the auditory–visual–tactile interface (205.53 ± 64.96) was significantly greater than that under the purely visual interface (118.67 ± 55.40). The maximum saccade velocity of the participants under the visual–tactile interface (169.90 ± 46.99) was significantly greater than that of participants under the auditory–visual interface (100.36 ± 39.07). The maximum saccade velocity of the participants under the auditory–visual–tactile interface (205.53 ± 64.96) was significantly greater than that of participants under the auditory–visual interface (100.36 ± 39.07).

#### 4.3.3. Comparison of Average/Maximum Saccade Amplitude under Different Warnings

A single-factor analysis of variance method was used to compare the effects of four multi-modal interactive combination warnings on the average/maximum saccade amplitude of participants during the takeover in the level 3 warning interface. It was found that there was a significant difference in the average saccade amplitude (*p* = 0.001). There was also a significant difference in the maximum saccade amplitude (*p* = 0.003).

Through multiple comparisons after ANOVA, it was found that the average saccade amplitude of the participants under the purely visual interface (4.68 ± 2.42) was significantly smaller than that under the auditory–visual–tactile interface (6.88 ± 2.21). The average saccadic amplitude under the auditory–visual interface (3.46 ± 46.99) was significantly lower than that under the auditory–visual–tactile interface (6.88 ± 2.21). The average saccade amplitude of the participants under the visual–tactile interface (4.61 ± 1.66) was significantly smaller than that of participants under the auditory–visual interface (6.88 ± 2.21) (Figure 4).

**Figure 5:**
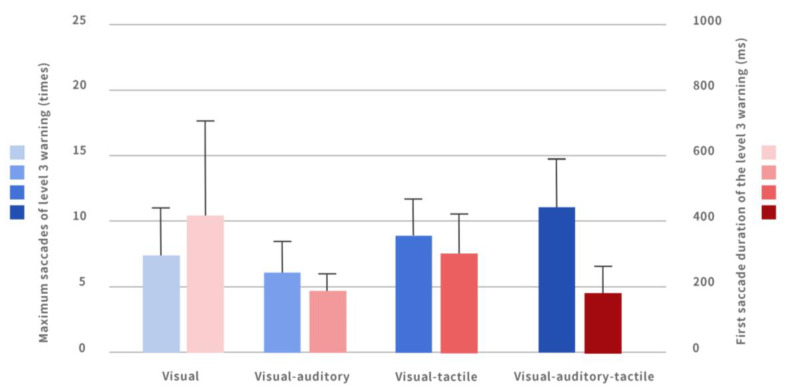
Maximum saccades and first saccade duration of level 3 warning interface under different warnings.

Multiple comparisons after ANOVA showed that the maximum saccade amplitude of participants under the purely visual interface (7.49 ± 3.27) was significantly lower than that of participants under the auditory–visual–tactile interface (11.18 ± 3.70). The participants’ maximum saccade amplitude under the auditory–visual interface (6.33 ± 1.67) was significantly smaller than that when using the auditory–visual–tactile interface (11.18 ± 3.70) (Figure 5).

#### 4.3.4. Comparison of the First Saccade Duration under Different Warnings

One-way ANOVA was used to compare the effects of four multi-modal warning modes on the first saccade duration of participants during the takeover in the level 3 warning. The results showed that there was a significant difference in the first saccade duration (*p* = 0.006).

By pairwise comparison, the participants’ first saccade duration under the auditory–visual interface (190.67 ± 54.13) was significantly shorter than that under the purely visual interface (411.15 ± 306.753). The first saccade duration under the auditory–visual–tactile interface (189.33 ± 76.32) was significantly shorter than that under the purely visual interface (411.15 ± 306.753) (Figure 5).

## 5. Discussion

The current level 3 visual interface of high-speed train warning has the most abundant graphic information among the three levels, but the abundant graphic information does not correspond to a better prompting effect. From the perspective of a strictly visual interface, there was no significant difference between the takeover times of the participants under the level 1 warning and the level 2 warning, but both times were shorter than the takeover time under the level 3 warning. However, it was found that first fixation durations in the level 2 warning and level 3 warning were significantly less than that in the level 1 warning, showing that the combination of graphics and text in the second warning and the third warning was more effective than the single text reminder in the first warning, and made it easier to attract the participants’ attention. Nevertheless, with excessive graphic information, the level 3 warning does not correspond to a better prompting effect. Combined with the results of takeover time, the level 3 warning has more complex warning signals and has the highest takeover time, indicating that the complex warning signals increase the understanding cost of participants. In addition, it was also found that the total fixation duration of participants in the level 3 warning situation is the highest among the three. Therefore, the graphic information of the warning should be simplified to help high-speed train drivers obtain takeover information more intuitively.

Compared with the visual interface of the level 1 warning, the level 2 warning visual interface of the current high-speed train is more likely to attract the attention of the drivers, but the relevance of and guidance between the graphic and text content can be further optimized. From the perspective of the total fixation duration, the total fixation duration of the level 1 warning in the screen interface is significantly smaller than that of the level 2 warning. From the viewpoint of eye movement trajectory, this may be because, in the level 2 warning interface, as the warning signs and text information are separated, the participants tend to look first at the warning sign and then focus on the text area so as to obtain information. In the level 1 warning, participants look directly at the text area to access the information and, as a result, the total fixation duration of the level 2 warning is significantly longer than that of the first warning. Moreover, this conjecture was also confirmed in the comparison of fixation counts in the region of interest. The fixation count in the level 2 warning was significantly greater than that in the level 1 warning. The fixation count is related to the efficiency of visual search, and the greater the fixation count the lower the efficiency of the search strategy [41]. Our results showed that the efficiency of visual access to information was relatively low under the level 2 warning. This phenomenon is similar to the difference in the distribution of fixation count for novice/skilled drivers found in other studies [42]. This indicates that, although the level 2 warning interface has good takeover time and attention-getting characteristics, it still needs to be improved in the aspect of image and text warning information fusion. Namely, in the optimization design of the high-speed rail warning interface, the strong reminder of the level 2 warning compared with the level 1 warning should be retained, and the relevance and guidance between the graphic–text design elements of the level 2 warning should be emphasized (such as adding arrows to visually guide the user). By optimizing the visual design of the level 2 warning, it will be possible to reduce the search path of high-speed rail drivers and reduce the total fixation duration under the level 2 warning.

Multi-modal warning interfaces have higher response efficiency than single-modal warning interfaces. By comparing the takeover times of participants under different warning interfaces, we found that the takeover time of all the multi-modal warning interfaces was lower than that of the purely visual interface. The first saccade duration of all the multi-modal warnings interfaces was lower than that of the purely visual interface. Therefore, compared with the purely visual interface interaction, the multi-modal warning interface has a higher response efficiency for high-speed train drivers.

The auditory–visual multi-modal interface is most suitable for the most urgent (level 3) high-speed train warning. It was found that the auditory–visual warning interface had the highest takeover efficiency among the four multi-modal interfaces, because the takeover time of the auditory–visual warning interface was the lowest, especially significantly lower than the current purely visual warning interface. In terms of saccade index, compared with other multi-modal interfaces, the auditory–visual warning interface has significant advantages in saccade count, maximum saccade velocity, and average/maximum saccade amplitude. This means that the addition of an auditory modal interface to the current purely visual warning interface can significantly improve the cognitive efficiency of high-speed train drivers and reduce the sense of urgency, which is contrary to the addition of tactile cues. Therefore, the auditory–visual multi-modal interface is the most suitable for the level 3 train warning, which is consistent with the research conclusion of De Groot et al. [28] in automobiles.

The introduction of an auditory interface can increase the efficiency of a purely visual interface, but the introduction of a tactile interface does not improve the efficiency. By comparing the effects of different multi-modal warnings on participants’ takeover time, we found that the takeover time of the auditory–visual warning interface was significantly shorter than that of the visual warning interface, while no significant difference was found between the other multi-modal interfaces and the purely visual interface. The tactile interface did not improve the driver’s takeover efficiency. This is different from the research conclusions of Prewett et al. [29] in automobiles. In the analysis of the saccade count of different multi-modal modes under the level 3 warning, it was found that the saccade counts of the two groups with a tactile modality were significantly greater than those of the other two groups in the process of taking over. In the analysis of maximum saccade velocity, the maximum saccadic velocity of the auditory–visual–tactile interface was significantly higher than that of the purely visual interface and the auditory–visual interface, and the maximum saccadic velocity of the auditory–visual–tactile interface was significantly higher than that of the auditory–visual–tactile interface. In the analysis of the average/maximum saccade amplitude, it was found that the average saccade amplitude and maximum saccade amplitude of the auditory–visual–tactile interface were significantly larger than those of the purely visual interface and the auditory–visual multi-modal interface. Through the above analysis, it can be found that the saccade count, velocity, and amplitude of participants with a tactile interface were significantly higher than those of participants without a tactile interface. In other studies, it has been found that, when the driver’s sweep is large, the participant may be nervous because of the tension generated, which also indicates that the driver’s recognition ability is in a poor state [40]. Based on this, the introduction of a tactile pattern warning interface did not have a positive oculomotor effect on participants and even resulted in reduced recognition ability due to tension. Therefore, the addition of a tactile mode interface does not improve driver pick-up efficiency.

## 6. Conclusions

To help improve the active safety of automatic driving high-speed trains, we conducted a high-speed train simulation driving experiment in this study. We compared and determined the best multi-modal warning interface form in the emergency takeover situation of automatic driving high-speed trains and put forward optimization and improvement suggestions for the existing automatic driving high-speed train warning interface. The results showed that the current level 3 visual interface of high-speed train warning had the most abundant warning graphic information, but this increase in graphical information does not increase the takeover efficiency of the driver. When optimizing the design of warning interfaces, graphic information should be simplified to help high-speed train drivers obtain takeover information more intuitively. Compared with the visual interface of the level 1 warning, the form of the level 2 warning visual interface is more likely to attract the attention of drivers, but it still needs to be optimized in terms of the relevance and guidance between graphic–text elements. The multi-modal alert interface has faster response efficiency than the single-modal alert interface. The auditory–visual multi-modal interface is the most efficient, and it is suitable for the most urgent (level 3) high-speed train warning. The introduction of an auditory interface can increase the efficiency of a purely visual interface, but the introduction of a tactile interface does not improve efficiency. The above results are helpful to improve the active safety of autonomous high-speed trains, which is of great significance to protect the health and safety of the public.

### Limitations

1. None of the participants in the study involved in the experiment were professional drivers with experience in driving high-speed trains. Driving experience may have an effect on learning performance and takeover time, so there are limitations to the experimental results, and professional high-speed train drivers will be introduced for experimental investigation in the subsequent study.
2. In the experiment, for the sake of experimental feasibility, the duration of simulated driving was set at 20 min and participants were asked to stay focused during the 20 min. In actual driving, the length of driving is usually one hour and above. The study was limited in its control of driving time and ignored the effect of driving fatigue on the driver taking over the operation.

## 7. Relevance to Industry

These findings can be used as a basis for the interface design of automatic driving high-speed trains and help to improve the active safety of automatic driving high-speed trains, which is of great significance to protect the health and safety of the public.

## Figures and Tables

**Table 1:** Automation level of high-speed train automatic driving system.

Automation Level	Name	Definition	Application Status
GOA1	Manual driving under the supervision of ATP (Automatic Train Protection)	All operations of the train are controlled by the driver, and the driver handles the emergency.	It has been widely used.
GOA2	Automatic driving with driver monitoring	Equipped with automatic driving system. The daily operation is controlled by the system, and a driver is on duty to handle emergency situations.	The high-speed train automatic driving system that is currently being developed and can be realized at present.
GOA3	Manned autonomous driving	Drivers are replaced by automatic driving system and other system functions, and only crew members are arranged to deal with emergencies.	Not yet applied.
GOA4	Unattended autopilot	At present, it is the highest level of train automation system. There are no drivers or crew members, and all functions are automatically managed by the system.	Not yet applied.

**Table 2:** High-speed train interface warning method.

Warning Level	Urgency Level	Interface Warning Method	Disposal of Drivers
Level 1	Low	Report warning information in text (Figure 1a).	Needs to be checked immediately, no need to be processed immediately.
Level 2	Medium	A red triangle icon is displayed in the upper-right corner of the screen, including a text report and warning identification (Figure 1b).	Needs to be checked immediately, can be processed immediately, or can be processed after arriving at the station.
Level 3	High	A red triangle icon is displayed in the upper-right corner of the screen, and warning information such as group number, carriage, warning type, fault code, and treatment measures are reported in the form of pop-up window (Figure 1c).	Needs to be checked immediately, needs to be processed immediately.

**Table 3:** Comparison of the takeover time, first fixation duration in AOI, total fixation duration in AOI, and the fixation count points in AOI under different warning levels and pairwise comparison.

	Takeover Time	*p*	Contrast Item	Adjusted Significance
level 1	1.863 (1.585–2.223)	<0.001	level 2	0.368
level 2	1.649 (1.468–2.01)		level 3	<0.001
level 3	2.794 (2.295–3.11)		level 1	<0.001
	First fixation duration within AOI	*p*	Contrast item	Adjusted significance
level 1	586 (327.5–891.5)	<0.001	level 2	0.118
level 2	267.5 (182.25–423.5)		level 3	<0.001
level 3	368.5 (271–545.75)		level 1	0.037
	Total fixation duration in AOI	*p*	Contrast item	Adjusted significance
level 1	392 (322.75–527.5)	<0.001	level 2	0.025
level 2	531.5 (422–667.25)		level 3	0.435
level 3	600.5 (426.75–854.25)		level 1	<0.001
	Fixation counts in AOI	*p*	Contrast item	Adjusted significance
level 1	2 (1–2.33)	0.001	level 2	1.000
level 2	2 (2–3)		level 3	0.006
level 3	1.67 (1.33–2)		level 1	0.002

## Data Availability

The datasets generated during and/or analysed during the current study are available from the corresponding author on reasonable request.
